# Laparo-Elytrotomy

**Published:** 1879-02

**Authors:** Thomas Whiteside Himes


### LAPARO-ELYTROTOMY.

By THOMAS WHITESIDE HIMES, B.A., M.B.

The following is the first case of laparo-elytrotomy which I have heard of being performed in Europe.

On Sunday afternoon, July 14th, I was summoned by one of the midwives attached to the Hospital for Women to visit Mrs. O'M——, who had been in labor (at full time) for over twenty hours, without any advance of the child. On entering the wretched house, situated in a court, I was almost overcome by a most offensive stench. It was so horrible that I asked if pigs were kept in the house, really believing it to be the case. I learned, however, that the unfortunate patient was the source of the fetor. I found her advanced in labor to the end of the first stage, the child being alive and in the first position, the head only entering the brim. The recto-vaginal septum was converted into a cancerous mass, compact, inelastic and rough, which extended from the perineum to within an inch of the posterior vaginal cul-de-sac. The upper part of the posterior and the anterior wall of the vagina were soft and elastic, and the uterus also was apparently quite healthy. The vagina was so narrowed by the new growth occupying its posterior wall that less than two inches remained for the passage of the child. Labor-pains had ceased entirely for over an hour.

*History.*—The patient was thirty-seven years of age, had been married ten years, and had borne nine children, pregnancy on each occasion lasting the full time. Four children still survived. During her whole married life she had been a rather heavy drinker, her habits in this respect being conformable to those of her husband. Her face and large fatty liver and heart gave evidence of this. Her last child was two years old. For about a year after its birth she became very stout, but subsequently she lost much flesh, and was, when seen, a thin, badly nourished woman. For the past eight months she had been suffering from excruciating pain in the rectum, with occasional attacks of severe hemorrhage, and for the last three months her motions had only come per vaginam. There was a constant and stinking discharge from the anus and vagina. For the last eleven weeks she had been unable to get out of bed, from debility and pain; and for over forty-eight hours preceding my visit she had been incessantly vomiting, being unable to retain anything in her stomach. For some days she had been suffering with diarrhœa. The heart's action was feeble and irregular.

It was evident that operative measures alone could effect the delivery of the child, which was still alive. The limited size of the vagina, which was scarcely two inches wide; the nature of the obstruction, its unyielding character and its disposition to hemorrhage, as evidenced by the serious losses of blood which had frequently occurred spontaneously; and the fact that the child's head had not passed the outlet, precluded operation by the vagina without fatal laceration. Having then just read the interesting paper by Dr. T. Gaillard Thomas, of New York, on laparo-elytrotomy, I determined to try it, as the state of the woman was so grave that Cæsarian section would evidently cause instant death. As the patient assented to my suggestion, I had her removed to the Hospital for Women. Owing to the day and the hour, I could only get the assistance of one of my colleagues at the hospital, and I had great difficulty in finding one other medical man at home to help me. At 6:30 P.M. I proceeded to operate, assisted by Drs. E. Jackson and O'Keefe. Had the case not been urgent, I would not have operated with only three pairs of hands, as one of them being necessarily occupied exclusively with the anæsthesia, the two others are insufficient in number (no matter how efficient) for what has to be done. But had the child descended into the pelvis, laparo-elytrotomy would probably have been rendered impossible, and fatal injury would very likely have been done. So further delay was unadvisable.

*Operation.*—I intended performing this operation under strict antiseptic precautions, as I am in the habit of doing when possible, but unfortunately the spray belonging to the hospital was at the time being repaired, and one which borrowed was not in good order. With the exception of the spray I employed antiseptic measures. The patient having been placed on the operating table and chloroformed, I made an incision through the abdominal wall in the direction of a line extending from the spina ilii ant. sup. sinistr. to the spina pubis. After a little difficulty in distinguishing a layer of fat which simulated the appearance of the omentum, the peritoneum was reached and readily recognized, being much more ample than in non-pregnant women, and hanging in folds at the bottom of the wound. I next passed a blunt probe up the vagina, and by it pushed the anterior vaginal cul-de-sac in the wound. Seizing this with a pair of hooked forceps, I divided it, and passing my finger through this orifice, felt the os uteri. Some slight difficulty was experienced at this part alone of the operation, owing to the small space which existed between the anterior surface of the enlarged uterus and the brim of the pelvis. Having extended the wound, I passed my hand through it into the fully-dilated os, which was occupied by the head and bag of waters. I at once seized a foot and turned, and delivered a living male child without the least difficulty, the placenta being d livered simultaneously. The uterus contracted rapidly, and there was no uterine hemorrhage. There was not over an ounce of blood lost in the operation, a couple of small arteries, which were divided in the incision, having been at once secured by torsion. In fact, few ordinary labors are completed with less loss of blood. The operation lasted a little over twenty minutes. The wound was washed with carbolic lotion (5 per 100), and closed with gut sutures, and antiseptic dressings applied. The patient was then put to bed with hot bottles, etc. She was very much exhausted, and twice during the operation her heart became ominously feeble. On partially recovering, she became most violent, throwing herself about, shouting and using most abusive language. Three persons could scarcely hold her down in bed. After half an hour she became calmer and more rational, and subsequently had some hot coffee and brandy (ether had been previously injected subcutaneously), which seemed to do her good. From time to time she again grew violent, but speedily sank exhausted, though sensible. She seemed to be rallying, when, after about two hours, she unexpectedly sat up in bed, but in a few minutes grew livid, faint, and sank dying. Artificial respiration was tried in vain for ten minutes, and other measures, but ineffectually, and she shortly expired.

*Necropsy.*—Next day I made an examination of the parts involved in the operation before the members of my class, going through the steps of the operation, for their benefit, on the right side of the body. A clot, about the size of a couple of walnuts, lay in the bottom of the wound. The bladder and peritoneum were quite uninjured, and all other parts except those intentionally incised. The uterus was well contractəd, quite healthy, and the os and cervix free from laceration. There was no trace of cancerous disease in the upper part of the vagina.

*Remarks.*—That the fatal termination of this case was not a thing to be surprised at will be the opinion of most readers, as well as that Cæsarean section would not have been more successful. My opinion is that the unavoidable hemorrhage, and the shock of cutting through the abdominal wall, laying bare the abdominal cavity, and dividing the uterine wall, would have caused the diseased and debilitated patient to die on the table. Had she been less violent, or strong enough to have justified one in giving morphia, or continuing the anæsthesia, her life might probably have been prolonged. That laparo-elytrotomy is a simple and feasible operation, I believe every reader will admit. It avoids almost all of the capital dangers of Cæsarean section, and is not more difficult. The wound is much less extensive, the peritoneum and uterus are not wounded at all, nor the abdominal cavity exposed to danger from infective fluids, cold, or mechanical injury; the danger of hemorrhage is much less, the shock is less, and the delivery of the child is quite as easy. As compared with craniotomy, this operation is simplicity itself, and the results hitherto obtained much better, being absolutely good for the child instead of absolutely fatal, and for the mother most salutary results have also ensued. Had the cancerous growth in this case been less extensive, so as to leave, say, a passage of three inches in the vagina, and had craniotomy been tried, would any better result have been probable for the mother after the deliberate destruction of the child? I scarcely think so. Craniotomy, when practiced with perfectly healthy tissues, is most unsatisfactory in its results for the mother, and I cannot but think that in such a case as this the unavoidable laceration of the diseased tissues which must have ensued, with consequent hemorrhage, the tediousness of the operation, and the shock incident on it, must have led to a fatal termination in so enfeebled a patient. Even supposing the peritoneal cavity should be accidently opened, the wound will be far less than in Cæsarean section, and the wound will be situated so as to favor, as far as possible, the escape of any blood, etc., from the abdominal cavity. Considering the easy nature of the operation, the certainty of saving the child, and the strong probability (judging from Dr. Thomas' report) of saving the mother, it is a question how far craniotomy will ever again be justifiable, and whether Cæsarean section should not drop into oblivion. I found my opinion more on the experience of Dr. Thomas and Dr. Skene than on my own single case. But the demonstration in this case of the truth and probability of what had been accomplished by the distinguished New York surgeons makes me think that the introduction (or revival) of this operation will exercise a great influence on operative midwifery in the future. The child is at present thriving.—*London Lancet.*

